# Swiss data quality: augmenting CAMELS-CH with isotopes, water quality, agricultural and atmospheric data

**DOI:** 10.1038/s41597-025-05625-1

**Published:** 2025-07-23

**Authors:** Thiago V. M. do Nascimento, Marvin Höge, Ursula Schönenberger, Sandra Pool, Rosi Siber, Martina Kauzlaric, Maria Staudinger, Pascal Horton, Marius G. Floriancic, Florian R. Storck, Päivi Rinta, Jan Seibert, Fabrizio Fenicia

**Affiliations:** 1 Eawag: Swiss Federal Institute of Aquatic Science and Technology, Dübendorf, Switzerland; 2 Department of Geography, University of Zurich, Zurich, Switzerland; 3 Geographisches Insitut, Universität Bern, Bern, Switzerland; 4 Oeschger Centre for Climate Change Research, University of Bern, Bern, Switzerland; 5 ETH Zürich, Zurich, Switzerland; 6 Hydrology Division, Federal Office for the Environment, 3003 Bern, Switzerland

**Keywords:** Hydrology, Environmental monitoring

## Abstract

Despite the growth of large-sample hydrology (LSH) datasets, water quality data remain scarce. Here, we introduce CAMELS-CH-Chem, an extension of CAMELS-CH (Catchment Attributes and Meteorology for Large-sample Studies in Switzerland), incorporating up to 40 water quality parameters for 115 Swiss catchments from 1981 to 2020. The dataset includes hourly and daily time series of measurements of water temperature, dissolved oxygen, pH, and electrical conductivity; as well as (bi)monthly measurements of dissolved organic carbon (DOC), total organic carbon (TOC), alkalinity (${{\bf{HCO}}}_{{\bf{4}}}^{{{-}}}$), ammonium (${{\bf{NH}}}_{{\bf{4}}}^{+}$), ${{\bf{NO}}}_{{\bf{3}}}^{{{-}}}$, ${{\bf{NO}}}_{{\bf{2}}}^{{{-}}}$, total nitrogen, dissolved reactive phosphorus, total filtered phosphorus, total phosphorus, Ca²⁺, Mg²⁺, Na⁺, K⁺, Cl⁻, ${{\bf{SO}}}_{{\bf{4}}}^{{\bf{2}}{{-}}}$, H_4_SiO_4_, ${{\bf{SO}}}_{{\bf{4}}}^{{\bf{2}}{{-}}}$, total hardness, and stream water isotopes. In addition, we provide catchment aggregated (bi)monthly time series of precipitation water isotopes, along with annual resolution data on land cover including specific agricultural information (crop types and livestock density), and atmospheric nitrogen deposition. This comprehensive dataset enables broader integration of water quality into LSH research and will support new insights specifically in the field of hydrological and biogeochemical modelling.

## Background & Summary

Recently, there has been a widespread development of large-sample hydrology (LSH) datasets worldwide^1–10^. Many of these datasets were inspired by the pioneering Catchment Attributes and MEteorology for Large-sample Studies (CAMELS) dataset, which provided a comprehensive LSH dataset for the contiguous United States^6^. Such datasets typically include hydro-climatic variables—such as streamflow, meteorological forcing data, and catchment properties (e.g., land use and soil types)—covering numerous catchments over extended time periods.

Yet, numerous studies have highlighted the importance of integrating long-term hydro-climatic and catchment properties with stream water quality data to derive critical insights into solute transport processes^11,12^. Hence, datasets that combine long-term, reliable water quality variables with other hydro-climatic and catchment properties are essential for investigating flow pathways and residence times, with practical applications in reducing pollutant loads and improving water resource management under pressures from population growth, land use intensification and climate change.

Recently, CAMELS-Chem^11^ was released as an openly accessible dataset for the contiguous United States—the first augmentation of a CAMELS dataset that incorporates water quality data. However, similar initiatives remain limited and freely accessible water quality parameters remain scarce in published datasets. This is primarily due to the challenges associated with measuring and providing access to such data, compared to hydro-climatic and catchment variables that are typically easier both to measure and to obtain.

Here, we introduce CAMELS-CH-Chem, an extension of the existing CAMELS-CH^5^ dataset. While CAMELS-CH provides hydrometeorological and streamflow data across Switzerland, CAMELS-CH-Chem builds on this by integrating up to 40 stream water quality parameters, isotopes and catchment-aggregated data on atmospheric deposition, land cover, and agriculture for 115 of the original CAMELS-CH catchments. With CAMELS-CH-Chem, we aim to make an important contribution to the field of LSH by introducing the first CAMELS extension in Europe to include water quality data, enabling data-driven modeling of water quality at larger scales.

Our intention in providing catchment-aggregated variables is to enable comprehensive, multi-scale analyses by offering a harmonized dataset ready for integration into LSH and hydro chemical modelling frameworks. Catchment aggregated data of agricultural practices and atmospheric deposition serve as critical explanatory variables for interpreting spatial and temporal variability in water quality and biogeochemical parameters. Similarly, the inclusion of precipitation and stream water isotope time series enables valuable insights into hydrological flow paths, water source contributions, and catchment-scale transit times.

Although some of the original data, dating back to 1970, is available upon request from providers such as FOEN^13^, CAMELS-CH-Chem specifically provides a comprehensive dataset from 1981 to 2020 to maximize the overlap between different water quality sources and the complementary CAMELS-CH. This approach aligns with the primary objective of LSH datasets: to provide long-term, standardized variables across large regions^14^.

The provided data are divided into three main categories:

(i) **Stream water chemistry:** This includes time series of more than 30 stream water chemistry constituents, covering both field and laboratory measurements. We provide hourly and daily data on water temperature, dissolved oxygen, pH, and electrical conductivity, along with (bi)monthly measurements of dissolved organic carbon (DOC), total organic carbon (TOC), alkalinity (${{\rm{HCO}}}_{3}^{-}$), ammonium (${{\rm{N}}{\rm{H}}}_{4}^{+}$), nitrate (${{\rm{NO}}}_{3}^{-}$), nitrite (${{\rm{NO}}}_{2}^{-}$), total nitrogen (TN), dissolved reactive phosphorus (DRP), total phosphorus (TP), total filtered phosphorus (TFP), calcium (Ca²^+^), magnesium (Mg²^+^), sodium (Na^+^), potassium (K^+^), chloride (Cl^−^), sulphate (${{\rm{SO}}}_{4}^{2-}$), silicic acid (H_4_SiO_4_), and total hardness. The choice of hourly and daily resolution aligns with the temporal structure of the CAMELS-CH dataset, while the (bi)monthly and other coarser intervals reflect the original sampling frequency provided in the respective data sources.
(ii) **Stream water isotopes:** This includes (bi)monthly time series of stream water isotope data of deuterium (²H) and oxygen-18 (¹⁸O).
(iii) **Catchment aggregated data:** This provides annual time series of atmospheric deposition concentrations for nitrate (${{\rm{NO}}}_{3}^{-}$), ammonium (${{\rm{N}}{\rm{H}}}_{4}^{+}$), ammonia (NH_3_), nitrite (${{\rm{N}}{\rm{O}}}_{2}^{-}$), and total inorganic nitrogen for 115 catchments, alongside land cover including specific information on crop type distributions and livestock density data. Finally, we also provide monthly time series of catchment aggregated precipitation isotope data to improve isotope assessments.

The manuscript is structured as follows: the Methods section describes the original data sources, and the methodology used for compiling it into the CAMELS-CH-Chem dataset. The Data Records section describes the structure of the CAMELS-CH-Chem dataset. Finally, the Technical Validation section provides a first order validation of the CAMELS-CH-Chem dataset based on selected hypotheses.

## Methods

### Stream water chemistry data

Water chemistry data were collected within the framework of national monitoring programs. The high-frequency water temperature data were obtained from the surface water temperature monitoring network of the Swiss Federal Office for the Environment (FOEN, in German BAFU). The water quality data were collected within the National Surface Water Quality Monitoring Programme (NAWA). Within NAWA FRACHT (previously called NADUF), the high-frequency parameters and pollutant loads are monitored in about 15 selected catchments in collaboration with the FOEN, the Swiss Federal Institute of Aquatic Science and Technology (Eawag) and the Swiss Federal Institute for Forest, Snow and Landscape Research (WSL). In the NAWA TREND, the surface water quality is monitored in more than 100 catchments in cooperation with the FOEN and the cantonal authorities. The three sources encompass both unique and redundant information for a few variables, whereby high-frequency data comprises the most complete daily and hourly time series.

**Fig. 1:**
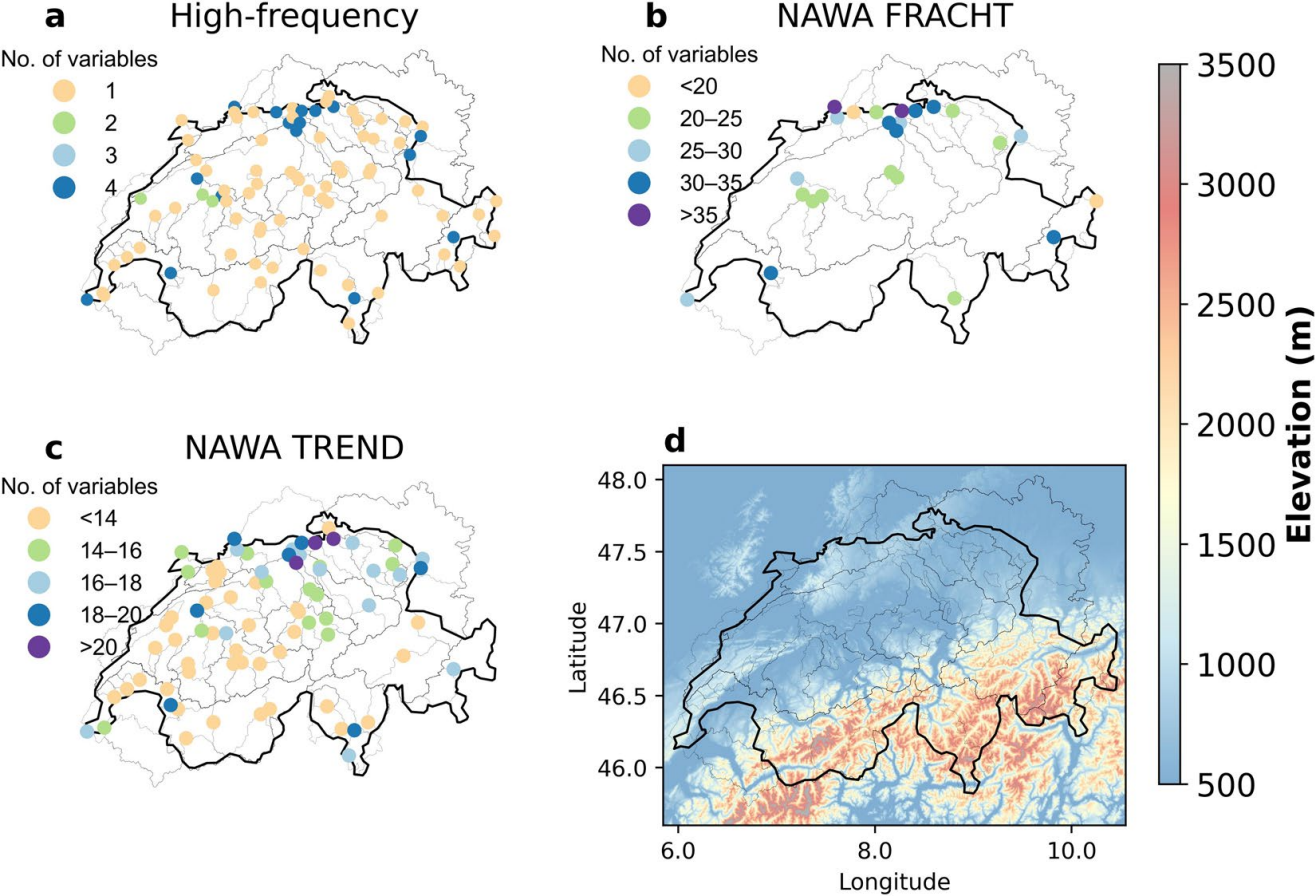
Spatial distribution of the measurement locations with data available in CAMELS-CH-Chem encompassing (**a**) high-frequency, (**b**) NAWA FRACHT and (**c**) NAWA TREND data. The dots in each subplot have different colors representing the number of available parameters at each location. Note that for the same location, we might provide data from high-frequency measurements, NAWA FRACHT and NAWA TREND. The upstream catchment area of each station is displayed in background. Additionally, subplot (**d**) shows a map of the elevation, with the boundaries of Switzerland and the catchment areas of each station.

Figure 1 shows the distribution of the stream water chemistry measurement stations across Switzerland with their respective catchment boundaries in the background. The colors of each dot represent the maximum number of chemical variables observed at a given location. Note that some locations of the three different data sources overlap, consequently we provide data for 115 unique locations. Figure 1 also illustrates that these catchments are well distributed across Switzerland capturing the complex topography (Fig. 1d) and climatology of the country. In total, 86 locations have high-frequency measurement data available, 24 have water sampling data from NAWA FRACHT and 76 from NAWA TREND.

In the following section, we describe the main data sources. A detailed description of the measurements and information on data acquisition and processing, such as sensor types, accuracy, and methods used, are available in Supplementary Material, i.e., Table S1 for the high-frequency data, Table S2 for NAWA FRACHT, and Table S3 for NAWA TREND.

### High-frequency (sensor) data

The FOEN^13^ and NAWA FRACHT^15^ programme provided hourly and daily time series of stream water temperature, pH, electric conductivity, and oxygen concentration from 1981 to 2020 for the 86 stations shown in Fig. 1a. It is important to note that most of the 86 stations have temperature only. These data are measured with online sensors (telemetry). Here we refer to them as “high-frequency data.” A further overview of these four variables regarding the dataset-specific information (name and description), units and resolution is shown in Table 1.

**Table 1:** Overview of the stream water chemistry data obtained from the high-frequency data provided by FOEN and the NAWA FRACHT programme.

Attribute	Description	Units	Temporal resolution	Source
date	Date of the measurement.	—	Hourly, daily	FOEN^13^ and NAWA FRACHT^15^
temp_sensor	Water temperature	°C		
ec_sensor	Electrical conductivity at 25 °C	μS/cm		
O2C_sensor	Oxygen concentration	mg/L		
pH_sensor	pH	—		

### Nawa fracht

Further water chemistry data were obtained from the NAWA FRACHT programme^15^ (Fig. 1b). The dataset provides 38 variables obtained from either installed online sensors (six variables with **“_sensor”** in their names) or measured in the laboratory (remaining variables) (Table 2). These data have a measurement resolution of 7 to 14 days collected between 1982 and 2020, whereby time series provide the mean of measurements between **date_start** and **date_end**. Note that the NAWA FRACHT program also has overlapping measurement locations with the high-frequency data (described above) and NAWA TREND (described below). An overview of the 38 measured variables, i.e., dataset-specific information, units, and resolution, is shown in Table 2.

**Table 2:** Overview of the stream water chemistry variables obtained from the National River Monitoring and Survey Programme (NAWA FRACHT, previously called NADUF).

Attribute	Description	Units	Temporal resolution	Source
date_start	Measurement start date	—	7–14 days mean	NAWA FRACHT^15^
date_end	Measurement end date	—		
alk	Alkalinity	mmol/L		
As	Arsenic	µg/L		
Ba	Barium	µg/L		
Br	Bromide	mg/L		
Cd	Cadmium	µg/L		
Ca	Calcium	mg/L		
Cl	Chloride	mg/L		
Cr	Chromium	µg/L		
Cu	Copper	µg/L		
doc	Dissolved Organic Carbon	mg/L		
drp	Dissolved Reactive Phosphorus	mg/L		
ec25_sensor*	Electrical conductivity at 25 °C	µS/cm		
ec20_lab	Electrical conductivity at 20 °C	µS/cm		
F	Fluoride	mg/L		
Fe	Iron	mg/L		
Pb	Lead	µg/L		
Mg	Magnesium	mg/L		
q_mean_sensor*	Mean discharge	m^3^/s		
Hg	Mercury	µg/L		
Ni	Nickel	µg/L		
NO3___N	Nitrate nitrogen	mg N/L		
O2C_sensor*	Oxygen concentration	mg/L		
O2S_sensor*	Oxygen saturation	%		
pH_lab	pH	—		
pH_sensor*	pH	—		
K	Potassium	mg/L		
H4SiO4	Silicic acid	mg/L		
Na	Sodium	mg/L		
Sr	Strontium	µg/L		
SO4	Sulphate	mg/L		
tfp	Total filtered phosphorus	mg/L		
th	Total hardness	mmol/		
tn	Total nitrogen	mg/L		
toc	Total organic carbon	mg/L		
tp	Total phosphorus	mg/L		
tss	Total suspended solids	mg/L		
temp_sensor*	Water temperature	°C		
Zn	Zinc	µg/L		

The time series provide the mean of measurements between **date_start** and **date_end**.

*These values are averages computed for the **date_start** and **date_end** measurement interval and derived from sensors installed at the measurement location with an original resolution of 10-minutes (FOEN and NAWA FRACHT).

### Nawa trend

Water chemistry data are also provided from the NAWA TREND programme^16^ (Fig. 1c). This dataset provides 22 variables (Table 3), measured from grab samples covering 2011 through 2020 at monthly resolution. Thus, the time series represents the measurement taken at the respective **date**. An overview of the 22 variables, including dataset-specific information, units, and resolution, is shown in Table 3.

**Table 3:** Overview of the stream water chemistry variables obtained from the National Surface Water Quality Monitoring Programme (NAWA TREND).

Attribute	Description	Units	Temporal resolution	Source
date	Date the measurement was taken	—	One grab sample per month, between 2011 and 2020	NAWA TREND^16^
NH4_N	Ammonium nitrogen	mg N/L		
Cl	Chloride	mg/L		
q_max_kanton	Daily maximum discharge measured or estimated from cantons	m^3^/s		
q_min_kanton	Daily minimum discharge measured or estimated from cantons	m^3^/s		
q_mean_kanton	Daily mean discharge measured or estimated from cantons	m^3^/s		
q_mean_sensor*	Mean discharge	m^3^/s		
doc	Dissolved organic carbon	mg/L		
drp	Dissolved reactive phosphorus	mg/L		
ec25_lab	Electrical conductivity at 25 °C measured in the lab	µS/cm		
ec25_sensor*	Electrical conductivity at 25 °C	µS/cm		
NO3_N	Nitrate nitrogen	mg N/L		
NO2_N	Nitrite nitrogen	mg N/L		
O2_lab	Oxygen concentration measured in the lab	mg/L		
O2_sensor*	Oxygen concentration	mg/L		
O2S _sensor*	Oxygen saturation	%		
pH_lab	pH measured in the lab	—		
pH___sensor*	pH	—		
temp_lab	Water temperature measured in the lab	°C		
temp_sensor*	Water temperature	°C		
turbidity_sensor*	Turbidity	NTU		
tn	Total nitrogen	mg/L		
tp	Total phosphorus	mg/L		

The measurements were taken as grab samples, typically once per month. The precise sampling dates are provided in the **date** column in the final dataset.

*These values are averages computed from sensors installed at the measurement location at a resolution of 10-minutes (FOEN^13^ and NAWA FRACHT^15^) for the respective measurement date.

### Stream water isotopes

**Fig. 2:**
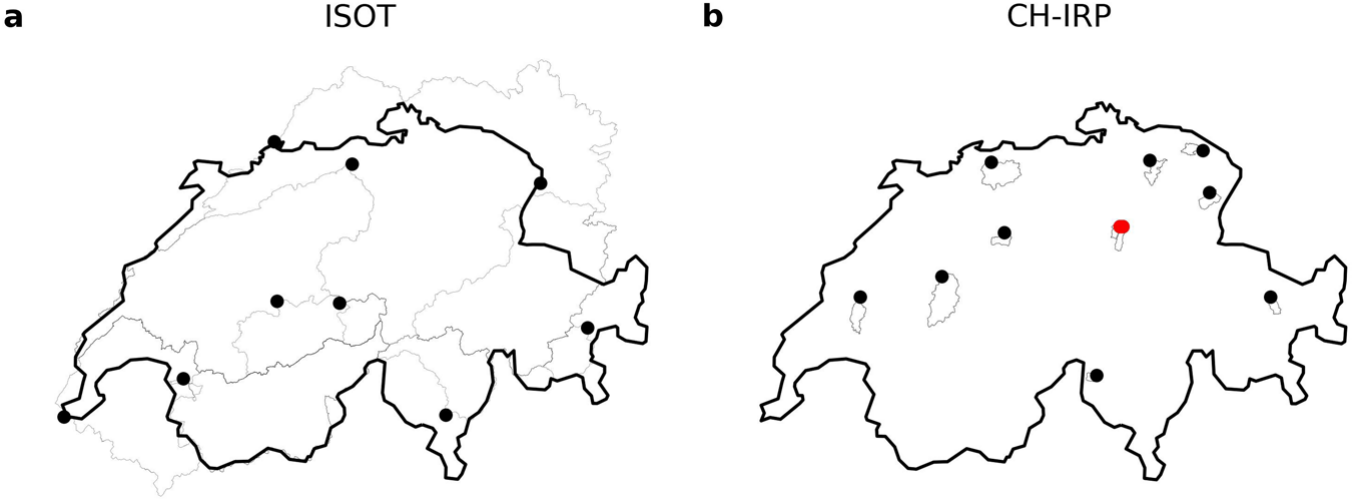
Spatial distribution of the measurement locations with stream water isotope data available in CAMELS-CH-Chem encompassing (**a**) ISOT data and (**b**) CH-IRP. Their respective catchment delineations are shown in background for both datasets. Moreover, for CH-IRP, it is worth noting that stations Biberbrugg (2604) and Einsiedeln (2609), both depicted as red dots, are located very close but in two different rivers (Biber and Alp). Therefore, due to scale reasons this figure gives the impression of having only 10 stations.

We also provide stream water measurements of deuterium and oxygen-18 data with a resolution from 14 days to monthly for all locations where such data are available across Switzerland from the ISOT module^17^ (nine stations) of the National Groundwater Monitoring (NAQUA) and from the CH-IRP dataset^18^ (11 stations). The spatial distribution of these stations is shown in Fig. 2.

### ISOT module

The ISOT module provides isotope measurement time series covering the period from 1992 through 2022. For two stations (i.e., Thun: ID 2030 & Brienzwiler: ID 2019), the measurements are derived from grab samples taken at the respective date. For the remaining stations, the values represent mixed samples aggregated between the start and end date. Information on the data is summarized in (Table 4). Figure 2a shows the distribution of these nine measurement locations.

**Table 4:** Overview of the ISOT isotope data available in CAMELS-CH-Chem.

Attribute	Description	Units	Temporal resolution	Source
date_start	Measurement start date.	—	Monthly or 14-day average	ISOT^17^
date_end	Measurement end date.	—		
delta_2h	Deuterium (^2^H)	δ ^2^H ‰ SMOW		
delta_18o	Oxygen-18 (^18^O)	δ ^18^O ‰ SMOW		

The resolution is variable for each time-step, and ranges from 14 days to monthly. The provided measurements are the average from daily samples between **date_start** and **date_end**, whereas aggregation intervals vary up to a maximum of 30 days.

### CH-IRP dataset

Apart from the nine stations monitored by the ISOT program (FOEN), here we also provide data for 11 stations monitored and made available through the CH-IRP dataset and project (Staudinger *et al*.^18^), covering the period from 2010 through 2020. The CH-IRP original dataset covers a total of 22 medium-sized alpine and pre-alpine Swiss catchments. It is worth noting that we deliberately provide data only for the 11 stations overlapping with the original CAMELS-CH stations. For a full description of the CH-IRP dataset, users should refer to their original publication (Staudinger *et al*.^18^). Information on the data is summarized in (Table 5), while Fig. 2b shows the distribution of these monitoring stations.

**Table 5:** Overview of the CH-IRP isotope data available in CAMELS-CH-Chem.

Attribute	Description	Units	Temporal resolution	Source
date	Measurement date.	—	Every 14-days	Staudinger *et al*.^18^
delta_2h	Deuterium (^2^H)	δ ^2^H ‰ SMOW		
delta_18o	Oxygen-18 (^18^O)	δ ^18^O ‰ SMOW		

These data are measured every 14 days. The **date** attribute corresponds to the sampling date.

### Catchment aggregated data

**Fig. 3:**
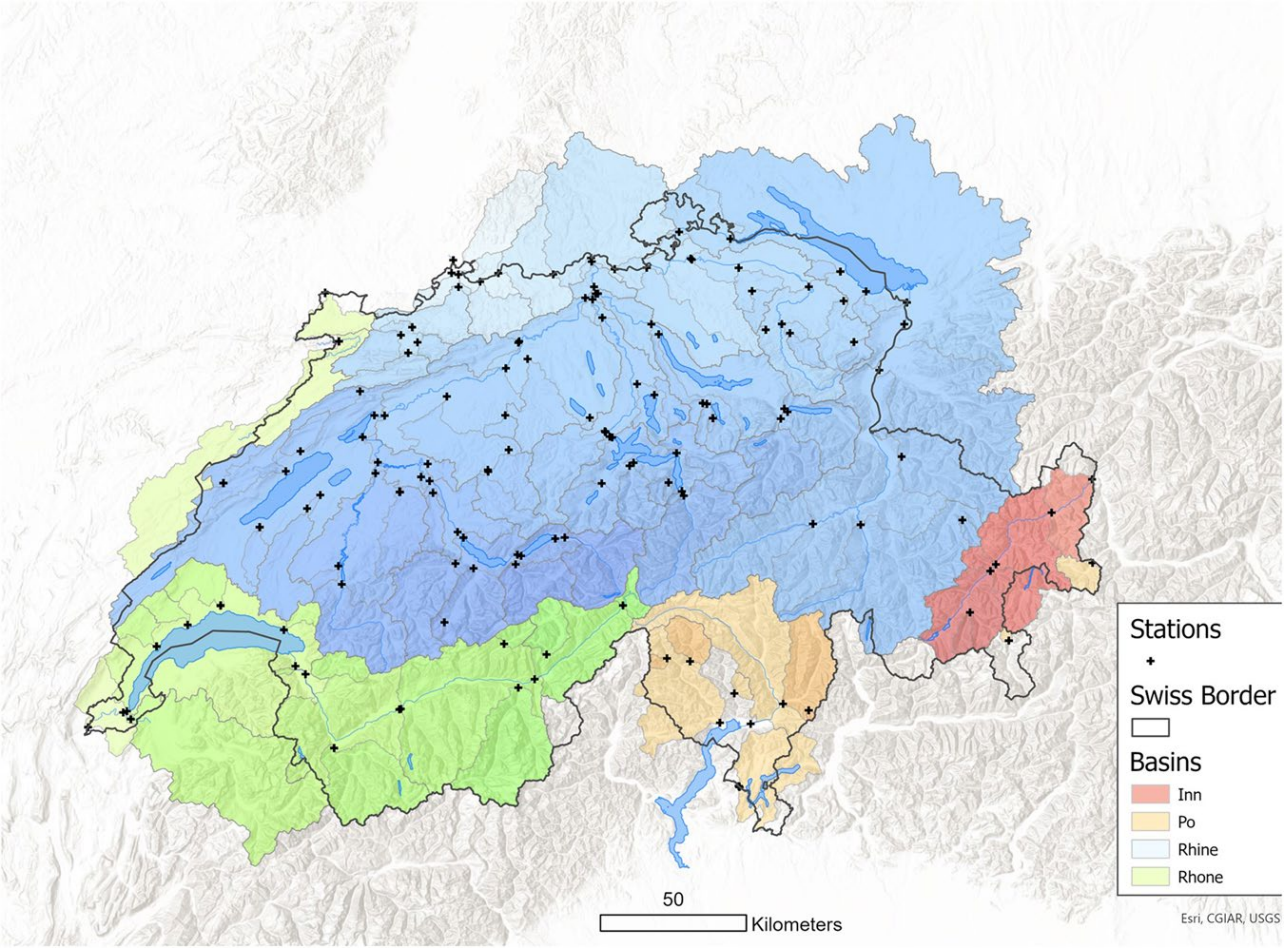
Spatial distribution of the 115 catchments used to derive the catchment aggregated data provided in CAMELS-CH-Chem. Each of their respective gauging stations is shown as black crosses. In the background, the four major basins are shown, along with their main river networks and major lakes.

Complementing the stream water chemistry and isotope data, CAMELS-CH-Chem also provides five types of catchments aggregated data: yearly time series of i) atmospheric deposition, ii) land cover percentage, iii) crop types and iv) livestock unit data, along with (v) monthly time series of precipitation isotopes data. Figure 3 shows the locations of the 115 catchments across Switzerland where these aggregated data are available. Note that these catchments correspond to those with stream water chemistry data.

It is important to note that, except for land cover data, this aggregated information was derived from data sources covering solely Switzerland. Since 23 of the 115 catchments have a part located outside Switzerland, users should be careful when dealing with the data. To provide an initial filter for users, the variable **area_perc_swiss**, which will be further described in the Gauge metadata section (Table 11), provides the percentage of the catchment located in Switzerland.

### Atmospheric deposition

CAMELS-CH-Chem also provides time series of annual atmospheric deposition of ${{\rm{N}}{\rm{O}}}_{3}^{-}$, ${{\rm{N}}{\rm{H}}}_{4}^{+}$, NH_3_, ${{\rm{N}}{\rm{O}}}_{2}^{-}$, HNO_3_, and total inorganic nitrogen aggregated from gridded data provided by Rihm and Künzle^19^. Specifically, the gridded data of individual nitrogen components was based on i) emission inventories and statistical dispersion models (NH_3_ and ${{\rm{N}}{\rm{O}}}_{2}^{-}$), ii) monitoring data and spatial interpolation methods (HNO_3_, wet deposition of ${{\rm{N}}{\rm{H}}}_{3}$ and ${{\rm{N}}{\rm{H}}}_{4}^{+}$), and iii) monitoring data, inferential models, and spatial interpolation (dry deposition of ${{\rm{NO}}}_{3}^{-}$ and ${{\rm{NH}}}_{4}^{-}$). Finally, the gridded data of total atmospheric nitrogen deposition are based on the combination of the above-mentioned components.

Further details on the methods used to model and spatially aggregate nitrogen deposition are available in Rihm and Achermann^20^ and Rihm and Künzle^19^. The original gridded nitrogen deposition dataset is available at a 1 km × 1 km resolution in 5-year intervals starting in 1990. Here, we provide catchment averages, which were calculated using the area-weighted mean of all map pixels inside a catchment. Table 6 provides an overview of this dataset. It is worth noting that since this dataset exists only for the years 1990, 2000, 2005, 2010, 2015 and 2020, we applied a linear interpolation to fill the years in between.

**Table 6:** Overview of the atmospheric deposition data available in CAMELS-CH-Chem.

Attribute	Description	Units	Temporal resolution	Source
date	Year of the measurement.	—	Yearly, between 1990 and 2020	^19,52^
dhno3gas	Gaseous deposition of HNO_3_	kg N/ ha		
dnh3gas	Gaseous deposition of NH_3_			
dnh4total	Sum of wet and dry deposition of ${{\rm{N}}{\rm{H}}}_{4}^{+}$			
dno2gas	Gaseous deposition of ${{\rm{N}}{\rm{O}}}_{2}^{-}$			
dno3total	Sum of wet and dry deposition of ${{\rm{NO}}}_{3}^{-}$			
dntotal	Total nitrogen deposition as a sum of wet, dry and gaseous deposition			

### Landcover data

We provide land use data for the 115 catchments included in CAMELS-CH-Chem, recomputed following the same procedure as in CAMELS-CH^5^, and also using the CORINE Land Cover (CLC) dataset^21^. As CLC data are available for the years 2000, 2006, 2012 and 2018, we applied linear interpolation to fill the years in between and repeated the value from 2018 for the two remaining years. The data were divided into 12 classes: agriculture, forest (coniferous, deciduous, and mixed), grass and herb vegetation, scrub vegetation, wetlands, ice and perpetual snow, inland water surface, rock (loose and solid), settlements/urban and unknown/blank. Table 7 summarizes this information. Users may refer to CAMELS-CH^5^ and the official CORINE^21^ publication for further details.

**Table 7:** Overview of the land cover data provided in CAMELS-CH-Chem.

Attribute	Description	Units	Temporal resolution	Source
date	Year of the measurement.	—	Yearly, between 2000 and 2020	CORINE^21^
crop_perc	Agriculture	%		
dwood_perc	Deciduous forest			
ewood_perc	Coniferous forest (evergreen)			
grass_perc	Grass and herb vegetation			
ice_perc	Glaciers and perpetual snow			
inwater_perc	Inland water			
loose_rock_perc	Loose rocks and bare soils			
mixed_wood_perc	Mixed forest			
rock_perc	Hard rocks and bare soils			
scrub_perc	Percentage of medium-scale vegetation			
urban_perc	Urban and settlements			
wetlands_perc	Wetlands			

The following two sections provide additional detail on agricultural practices within the “agriculture” land cover class described here. Specifically, the crop types and livestock density data represent subsets of this broader category, offering more granular insight into how agricultural land is used within each catchment.

### Crop type data

We also estimated the area within the 115 catchments covered by certain crops and provided these data annually from 1980 to 2020. The following 10 crop types were considered: cereals, maize, sugar beet, potatoes, rapeseed, pulses, vegetables, total arable land (=sum of all crops), as well as grapevines and orchards. We utilized the Swiss census of agricultural farms, provided by the Swiss Federal Statistical Office (FSO^22^), for the annual statistics of all crops in Switzerland.

Until 2019, yearly crop statistics were recorded only at the municipal level, meaning the precise location of crops within each municipality was unknown. Hence, to improve spatial localization, we distributed the statistical crop data by aggregating such yearly values across the land use class 41 (period 2004-09, and standard nomenclature NOAS04) obtained from the *Arealstatistik Schweiz* dataset^23^ for the arable land; and the classes “grapevine” and “orchard” from the Topographic Landscape Model (TLM) from the Swisstopo dataset^24^. This step resulted in the total crop data being divided into 10 different classes for each Swiss municipality.

In the end, we aggregated the municipality data per catchment and estimated the area of each crop type for each of the 115 catchments. Each catchment has, therefore, a yearly time series for each of the 10 crop classes (Table 8). It is important to note that the data before 1996 was provided at 5-year intervals (1980, 1985, 1990 and 1996), and after 1996, at yearly time-steps until 2019. Therefore, we applied a linear interpolation between the 5-year data in the 1980–1996 period and repeated the values from 2019 for the last year.

**Table 8:** Overview of the crop type data available in CAMELS-CH-Chem, along with their respective temporal resolution and source.

Attribute		Units	Temporal resolution	Source
date	Year of the measurement.	—	Yearly, between 1980 and 2020	^22–24^
Arable land	cereal	m^2^		
	maize			
	sugarbeet			
	potato			
	rapeseed			
	pulse			
	vegetable			
	total_arable			
grapevine				
orchard				

It is worth noting that the **crop_perc** attribute from the CORINE land cover dataset represents a broader coverage of agricultural land, including areas not covered by the specific crop types described here. Therefore, the sum of the individual crop type areas will typically be smaller than the corresponding agricultural land cover class from CORINE.

### Livestock unit data

The term livestock unit (here referred to as GVE, from the German word *Grossvieheinheiten*) is a reference unit that facilitates the aggregation of livestock across different species and age groups based on a standardized convention from Eurostat^25^. Here, we used yearly livestock unit data from the FSO^23^, covering the years 1980 to 2020. The original data were recorded at the municipal level, meaning the exact location of livestock within a municipality could not be determined.

Therefore, to improve spatial localization, we distributed the livestock data across the land use categories alpine and jura pastures, natural meadows, and farm pastures within each municipality. This was done by using land use classifications from the *Arealstatistik Schweiz* dataset^26^, allowing us to estimate livestock density (livestock units per hectare) for different land use types, including natural meadows, pastures, and Alpine and Jura pastures.

We distinguished between two types of areas:

1. Alpine and Jura Pastures: It is estimated that 20% of the total Swiss livestock population spends three months annually on these pastures. To calculate livestock density in these areas, we multiplied the total Swiss livestock units by 0.05 (20% × 1/4 year). This value was then divided by the total area of Alpine and Jura pastures, resulting in a uniform livestock unit per hectare for all such pastures.
2. Natural Meadows and Farm Pastures: We used land use categories 15 (natural meadows) and 16 (home pastures) from the Swiss land use statistics^23^. Each area was assigned a weighting factor of 1 and multiplied by the total livestock unit of the respective municipality. The resulting value was then multiplied by 0.95 (i.e., 1-0.05) and divided by the total area of natural meadows and home pastures within the municipality, yielding a municipality-specific livestock unit per hectare.

Finally, we aggregated the livestock data for the 115 catchments. An overview of the final livestock unit data is presented in Table 9. It is important to note that similarly to the crop-types data, livestock unit data before 1996 were provided at 5-year intervals (1980, 1985, 1990 and 1996) and after 1996, at yearly time-step until 2020. Therefore, we applied a linear interpolation between the years 1980 and 1996.

**Table 9:** Overview of the livestock unit data (GVE) available in CAMELS-CH-Chem, alongside their description, units, temporal resolution, and sources.

Attribute	Description	Units	Temporal resolution	Source
date	Year of the measurement.	—	Yearly, between 1980 and 2020	^23,25,26^
gve_sum	Number of livestock units per catchment.	unit		
gve_ha	Number of livestock units per hectare.	unit/ha		

Note that from 1980 to 1990, data are provided every five years, and from 1996 to 2020, data are provided yearly.

### Precipitation isotopes data

Stable isotopes of oxygen (^18^O) and deuterium (^2^H) in precipitation and in stream water serve as natural tracers of hydrological processes. Hence, besides the stream water isotopes time series (previously described), we provide monthly catchment-aggregated precipitation isotopes for the 115 catchments from 2007 to 2020.

In Switzerland, stable isotopes of oxygen in precipitation are monitored through the ISOT^17^ observation network, which is part of the NAQUA National Groundwater Monitoring Programme. Here, we used monthly precipitation isotope values from the ISOT network^27^, which were originally spatially interpolated into gridded isotope maps (“isoscapes”) using a regression-kriging approach^28^.

According to the isoscapes publication^27^, this interpolation method involves a multiple linear regression model relating isotope values to a set of geographic and climatic variables, including elevation, coordinates, and monthly precipitation totals. The spatially correlated residuals from this regression are then interpolated using ordinary kriging to account for local deviations not explained by the predictors. It is worth noting that the elevation dataset used originally had a resolution of 25 m (Swisstopo DHM25^29^) and was resampled to 500 m to match the final resolution of the isoscapes, serving as a compromise to align with the coarser resolution of the other input variables.

### Catchment delineation

We used the catchment boundary shapefiles from the CAMELS-CH dataset to calculate catchment aggregated data (e.g., atmospheric deposition and agricultural data). Catchment outlets in CAMELS-CH are defined based on the discharge gauging location. However, some chemical measurement locations in the NAWA FRACHT and NAWA TREND datasets are slightly different from the CAMELS-CH streamflow gauging stations. For these cases, we adjusted the CAMELS-CH catchment areas using the new outlet information. Information regarding these shifts is provided in the gauge metadata (Table 11) with details regarding the distance between streamflow and the water chemistry measurement locations. For the respective catchments, we also provide the adjusted shapefile delineation for users to decide whether to use the original CAMELS-CH or the adjusted CAMELS-CH-Chem catchment boundaries. All the remaining catchment aggregated data were derived exclusively using the catchment boundaries provided by CAMELS-CH.

## Data Records

The current version of the CAMELS-CH-Chem dataset (v1.0)^30^ is stored in a Zenodo repository at 10.5281/zenodo.16158375. The repository is organized into the following (sub)folders:

- ***catchment_aggregated_data:*** contains five subfolders. Each contains one csv file per catchment, with 115 files in total. The files are organized by time series (rows) and attribute variables (columns).
  - ***agricultural_data*:** contains one csv file per catchment with the variables described in Table 8.
  - ***atmospheric_deposition:*** like the previous, but with the variables described in Table 6.
  - ***landcover_data:*** like the previous, but with the variables described in Table 7.
  - ***livestock_data*:** like the previous, but with the variables described in Table 9.
  - ***rain_water_isotopes*:** like the previous, but with the variables described in Table 10.

**Table 10:** Overview of the isoscapes precipitation isotope data available in CAMELS-CH-Chem.

Attribute	Description	Units	Temporal resolution	Source
date	Measurement date.	—	Monthly between 2007 and 2020	Isoscapes^27^
delta_2h	Deuterium (^2^H)	δ ^2^H ‰ SMOW		
delta_18o	Oxygen-18 (^18^O)	δ ^18^O ‰ SMOW		

The data are provided at a monthly resolution. The **date** attribute is the sampling date.
- ***shapefiles:*** contains three subfolders.
  - ***camels_ch_del***: contains two shapefiles. One shapefile marks the location of the gauge stations (as will be described in Table 11), and the other includes the derived catchment boundaries associated with each gauge (Table 12). Both files are referenced in the Swiss coordinate system LV95 (sometimes also referred to as CH1903+) and were copied from the original CAMELS-CH.

**Table 11:** Overview of the gauges metadata structure with their respective variables name, description, and units.

Attribute name	Description	Units
gauge_id*	Catchment identifier according to FOEN notation.	—
sensor_id	The same as **gauge_id** for stations where water chemistry measurements from high-frequency measurements data are available	—
nawaf_id	Catchment identifier according to NAWA FRACHT notation.	—
nawat_id	Catchment identifier according to NAWA TREND notation.	—
isot_id	Catchment identifier according to ISOT notation.	—
chirp_id	Catchment identifier according to CH-IRP notation.	
gauge_name*	Gauging station name.	—
water_body_name*	Water body name.	—
gauge_easting*	Gauging station easting.	m
gauge_northing*	Gauging station northing.	m
gauge_lon*	Gauging station longitude.	°
gauge_lat*	Gauging station latitude.	°
area*	Catchment area derived using the FOEN outlet.	km^2^
area_swiss_perc	Percentage of the upstream catchment area located in Switzerland. A value of 100 means that the catchment is located completely within Swiss borders.	%
Q	Information if discharge time series from CAMELS-CH is available.	yes/no
level	Information if water level time series from CAMELS-CH is available.	yes/no
gauge_name_nawaf	Gauging station name according to NAWA FRACHT.	—
gauge_easting_nawaf	Gauging station easting according to NAWA FRACHT.	m
gauge_northing_nawaf	Gauging station northing according to NAWA FRACHT.	m
area_nawaf	Catchment area derived using the NAWA FRACHT sampling location.	km^2^
foen_nawaf_dist	Distance between the gauging station from CAMELS-CH and the NAWA FRACHT sampling location (0 when both are at the same location).	km
gauge_name_nawat	Monitoring site name according to NAWA TREND.	—
gauge_easting_nawat	Monitoring site easting according to NAWA TREND.	m
gauge_northing_nawat	Monitoring site northing according to NAWA TREND.	m
area_nawat	Catchment area derived using the NAWA TREND sampling location.	km^2^
foen_nawat_dist	Distance between the gauging station from CAMELS-CH and the NAWA TREND sampling location (0 when both are at the same location).	km
q_nawat_corrector	Weighting factor available to adjust the streamflow time series to the NAWA TREND catchment area.	—
remarks		—

*This information is the same as already provided in CAMELS-CH^5^.

**Table 12:** Catchment delineation metadata structure with their respective variable name, description, and units.

Attribute name	Description	Units
gauge_id	Catchment identifier according to FOEN notation.	—
sensor_id	The same as **gauge_id** for stations where water chemistry measurements from high-frequency measurements data are available.	—
nawaf_id	Catchment identifier according to NAWA FRACHT notation^16^.	—
nawat_id	Catchment identifier according to NAWA TREND notation^17^.	—
isot_id	Catchment identifier according to ISOT notation^18^.	—
gauge_name	Gauging station name	—
water_body	Water body name.	—
gauge_east	Gauging station easting.	m
gauge_nort	Gauging station northing.	m
gauge_lon	Gauging station longitude.	°
gauge_lat	Gauging station latitude.	°
area	Catchment area derived using the FOEN outlet.	km^2^
area_swiss	Percentage of the upstream catchment area located in Switzerland. A value of 100 means that the catchment is located completely within Swiss borders.
  - ***nawa_fracht_del***: provides the alternative delineation shapefile for the NAWA FRACHT catchments.
  - ***nawa_trend_del***: like the previous one, but for the NAWA TREND catchments.
- ***gauges_metadata:*** contains one csv file covering all the metadata associated with each of the 115 gauging stations, as will be described in Table 11.
- ***stream_water_chemistry:*** contains two subfolders.
  - ***timeseries*:** contains two nested sub-sub folders. The csv files in both are organized by time series (rows) and attribute variables (columns), and each column represents one of the four water quality variables as described in Table 1. Both nested subfolders contain 86 files.
    - ***daily***: contains one csv file per catchment at daily resolution.
    - ***hourly***: contains one csv file per catchment at hourly resolution.
  - ***interval_samples*:** contains two nested sub-subfolders.
    - ***nawa_fracht***: contains one csv file per catchment covered (24 files). The rows represent the dates, and each column represents one of the water quality variables, as described in Table 2.
    - ***nawa_trend***: contains one csv file per catchment covered (76 files) and presents a similar structure as the previous one, but now with each column covering one of the variables in Table 3.
- ***stream_water_isotopes:*** contains two subfolders. Each contains one csv file per catchment with any isotope data. The rows represent the dates, and each column represents either deuterium or oxygen-18 data.
  - ***isot:*** contains one csv file per catchment covered (nine files), as described in Table 4.
  - ***ch-irp:*** contains one csv file per catchment covered (11 files), as described in Table 5.

- ***additional_data_from_studies:*** contains a non-exhaustive overview of recent published studies performed in Switzerland^18,31–42^, where water quality and isotope measurements were monitored in catchments nested within the CAMELS-CH-Chem catchments. The data cover different periods of time, have different time resolutions, and occasionally provide also data from groundwater or snow samples. In the table reported in the csv file we recapitulate the available data, their resolution and the IDs within which the nested catchments are located. When data were not published, they were provided by the authors^31,32^, and are provided in the folder. For a complete description of the data, users are invited to refer to the corresponding studies.

### Gauge metadata

The gauge metadata file contains the basic information to allow a proper use of the dataset. Many attributes are a repetition of those provided by CAMELS-CH. Note that the coordinate information on northing and easting is always provided in the Swiss reference system LV95, while the **gauge_lon** and **gauge_lat** are provided in WGS84. Additionally, due to the potential location difference between the measurement point of the CAMELS-CH streamflow gauge and both NAWA FRACHT and NAWA TREND, further fields were added to ensure consistency when using the data.

The attributes **gauge_name_{},** **gauge_easting_{},** **gauge_northing_{}** and **area_{}** refer to specific information from either NAWA FRACHT or NAWA TREND when applicable. The attribute **area_swiss_perc** represents the percentage of the upstream catchment area located in Switzerland and might be useful for users when using the catchment aggregated data.

The field **foen_{}_dist** represents the distance in kilometres between the CAMELS-CH streamflow gauge and the NAWA FRACHT or NAWA TREND measurement points (when applicable). Additionally, we also added a correction factor (**q_nawat_corrector**) for the NAWA TREND measurement points, which can be used to correct the streamflow discharge (as provided in CAMELS-CH) to the new catchment area when using the chemistry data. Finally, the field **remarks** summarize additional potential information about the gauges that should be considered before using the data.

### Catchment delineations metadata

The delineated geometry of each catchment is stored in the catchment layer. This layer includes the **gauge*_*id** field, which is also used for the gauges, allowing for a link between the two datasets. Additionally, the catchment layer also includes the information shown in Table 12. These information ensure consistency between the catchment and gauge datasets, facilitating seamless integration and analysis.

## Technical Validation

### Calibration of the sensor, NAWA FRACHT and NAWA TREND data

The devices used to measure the variables available from sensor data are calibrated twice per year. If there are significant deviation from manual measurements, they are corrected accordingly against grab samples. For NAWA FRACHT, calibration of physico-chemical sensors at stations is performed monthly. More information about instruments accuracy and methods are available for both datasets in Tables S1, S2.

Regarding NAWA TREND, the data are measured, processed, and validated by either their respective cantonal authorities or their contracted laboratories. Although, we do not provide further details regarding the specific instruments, methodologies, or protocols used by the individual cantons or laboratory team, all the data is expected to be collected in accordance with the Swiss Modular Stepwise Procedure^43^ and analyzed using the methods recommended by the Swiss Lab’Eaux^44^.

### Water chemistry measurements first “sanity check”

We provided a first assessment of the validity of some of the measured variables. Based on previous literature, we formulated three main hypotheses on the expected variable correlations among themselves. We then tested these hypotheses to determine whether the observed water chemistry measurements are consistent with expectations. Here we computed the correlations using the Spearman correlation coefficient (*r*_*s*_). Our hypotheses are as follows:

(i) Stream water EC should be broadly negatively correlated with mean discharge^45,46^.
(ii) Conductivity measures the ability of the stream water to conduct electricity, which is directly correlated to the amount of dissolved ions^47,48^. Therefore, EC should be positively correlated to the measurements of major anions, such as Cl^−^ and ${{\rm{NO}}}_{3}^{-}$.
(iii) Increasing temperature decreases the solubility of oxygen in water, moreover, the increase in water temperature leads to an increase in biological activity, which can consequently reduce the concentrations of dissolved oxygen in the stream water^49,50^. Hence, stream water temperature and oxygen concentration should be negatively correlated.

Therefore, we selected the variables: ec25_lab, Cl, NO3_N and q_mean_sensor from NAWA TREND; ec20_lab, Cl, NO3_N and q_mean_sensor from NAWA FRACHT; and temp_sensor, and O2C_sensor from the high-frequency measurements data to perform our validation.

**Fig. 4:**
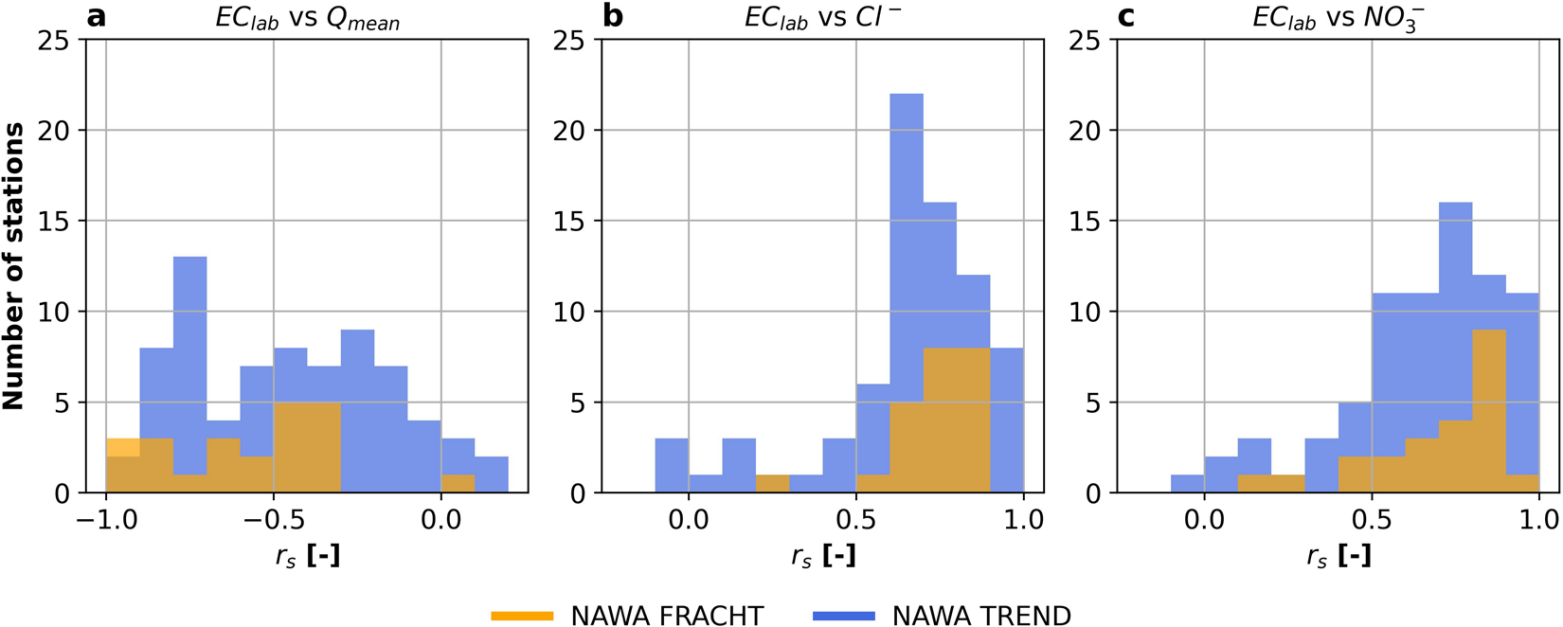
Histograms of the Spearman correlation coefficient between (**a**) EC_lab_ and Q_mean_, (**b**) EC_lab_ and Cl^−^, and (**c**) EC_lab_ and ${{\rm{NO}}}_{3}^{-}$. The different colors in the subplots represent different data sources, i.e., NAWA FRACHT in orange and NAWA TREND in blue.

Figure 4 shows the histograms of the distributions of the Spearman correlation coefficients (*r*_*s*_) computed between electrical conductivity and either mean discharge (a), Cl (b) and NO3_N (c) for NAWA TREND and NAWA FRACHT data. Overall, the correlations between electrical conductivity and mean discharge in Fig. 4a were largely negative, with values of *r*_*s*_ close to −0.50 for the three data sources. NAWA FRACHT only had one station out of 24 with a positive value, while there were 7 out of 76 for NAWA TREND. These findings are aligned with our hypothesis (i).

Moreover, Fig. 4b shows histograms for the correlations between electrical conductivity and Cl^−^, while Fig. 4c shows the histograms for the correlations between electrical conductivity and ${{\rm{NO}}}_{3}^{-}$. Both subplots show that most of the stations exhibit a correlation above 0.50, which supports our expectation from hypothesis (ii).

**Fig. 5:**
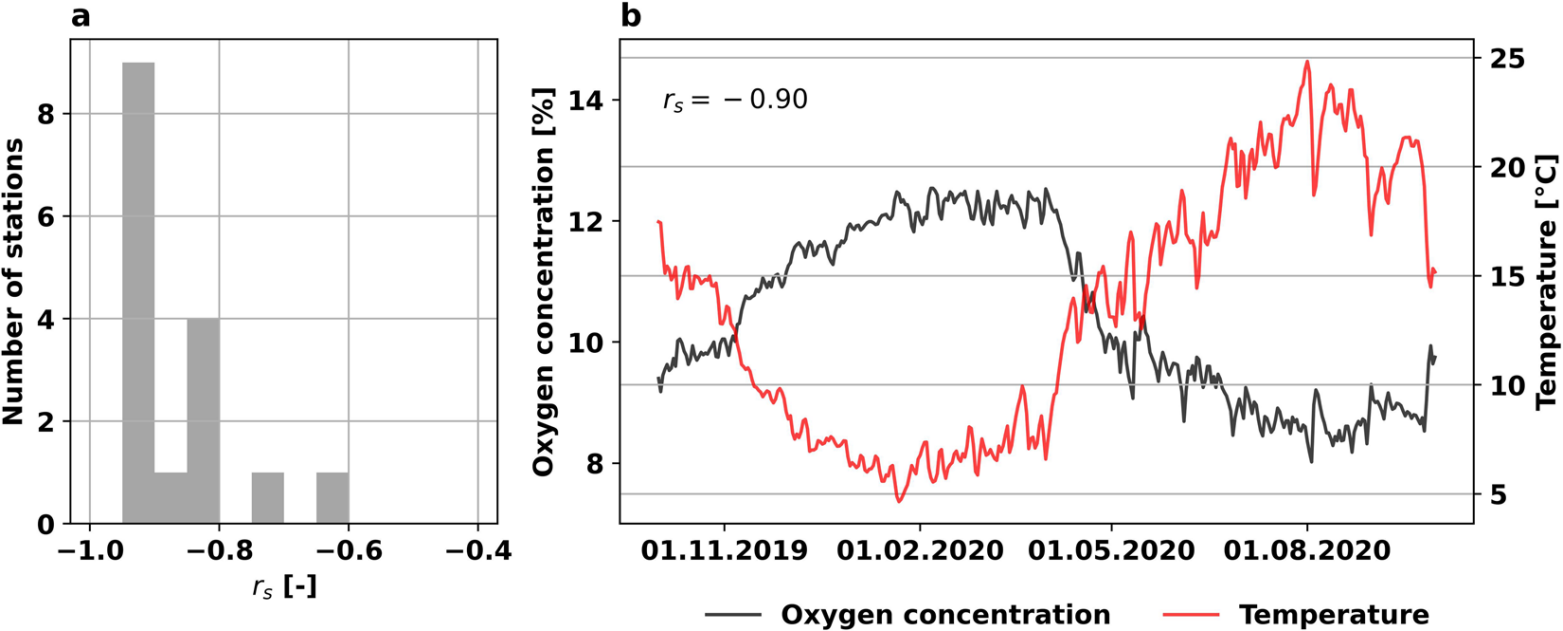
(**a**) Histograms of the Spearman correlation coefficient between the time series of oxygen concentration and temperature for all 16 stations covered by the high-frequency data. (**b**) Daily time series of oxygen concentration and temperature between 01.10.2019 and 30.09.2020 for gauge Mellingen (2018) used as an example of the typical annual course of the two variables.

Figure 5 shows the correlations between temperatures and oxygen concentrations. Figure 5a shows the histogram of the *r*_*s*_ computed for the daily time series of oxygen and temperature for each of the 16 stations with high-frequency measurement data for these two variables (Table 1). All correlations were negative, with only one station with *r*_*s*_ > −0.70. Figure 5b shows an example of a daily resolution time series of these two variables for the Mellingen gauge (2018) between 01.10.2019 and 30.09.2020. The figure indicates the expected pattern for the two variables, with oxygen concentrations increasing during the colder months and decreasing with rising temperatures during the summer period. Hence, these results corroborate our hypothesis (iii), which suggested a negative correlation between these two variables.

Overall, the rough confirmation of the three hypotheses stated in this section can be used as broad indication of the reliability of the current water chemistry datasets provided in CAMELS-CH-Chem. We acknowledge that this section does not contain a complete validation of the dataset, yet we believe that it is sufficient as a first sanity check of the overall validity of CAMELS-CH-Chem data.

### Ionic mass balance for NAWA FRACHT stations

**Fig. 6:**
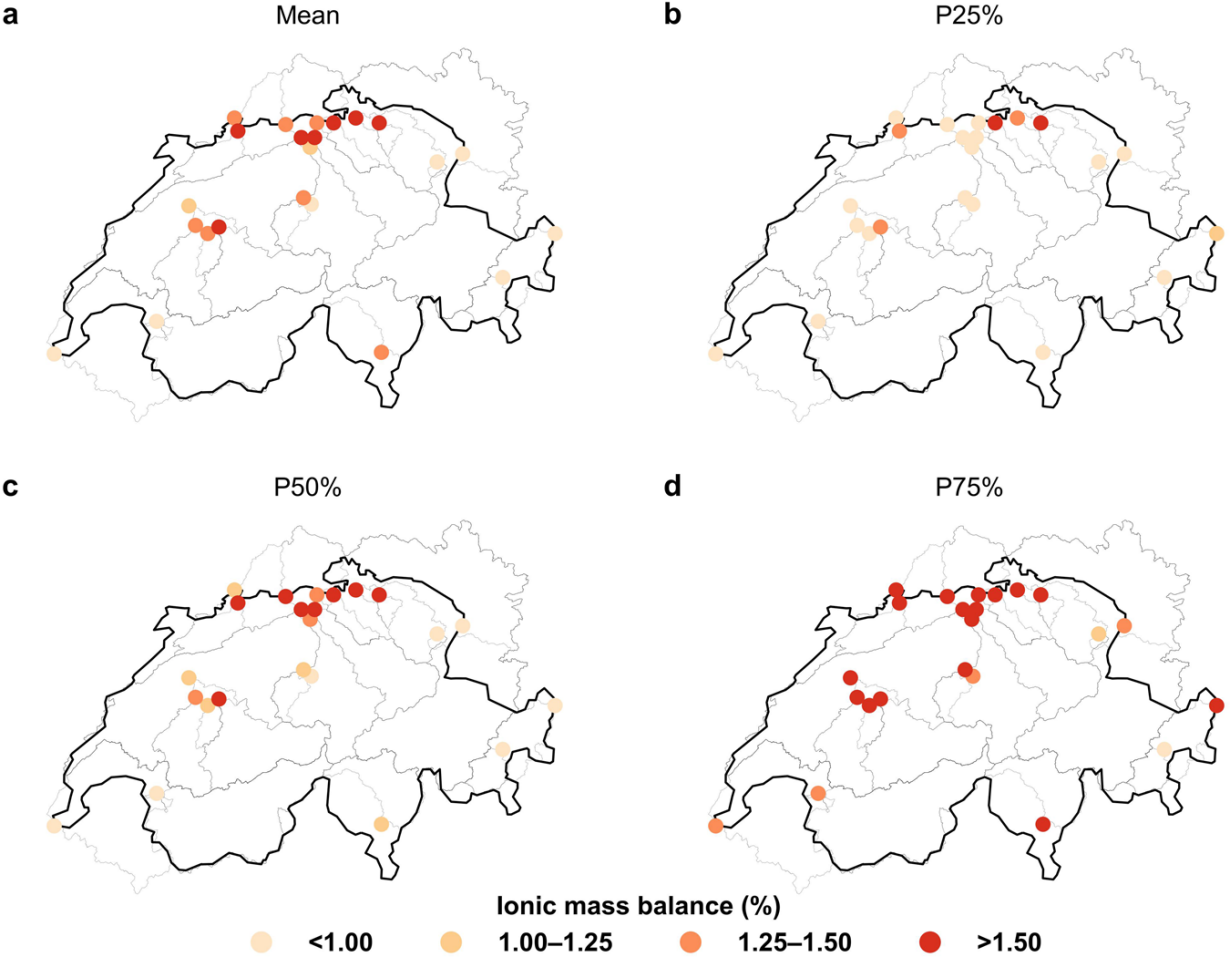
Distribution of the long-term (**a**) mean, (**b**) 25^th^ percentile, (**c**) 50^th^ percentile, and (**d**) 75^th^ percentile ionic mass balance for the NAWA FRACHT stations.

In this section we present the mean, and the 25^th^, 50^th^ and 75^th^ percentiles of ionic mass balance for all 24 NAWA FRACHT stations, based on the computation of the ionic mass balance for each measurement. Detailed information about the respective ionic balance of each station is available in Table S4 (**Supplementary Material**). Figure 6 shows the spatial distribution of these values.

About 70% of the stations presented a mean ionic balance below 1.5%, and a maximum 75^th^ of 4.3%. Additionally, only two stations presented a maximum ionic balance above 10% (2143: 14.8%; and 2415: 11%), although both their 75^th^ were below 4.3%, which indicates that such cases correspond to outliers in the stations (Table S4). Established literature^51^ suggests that values below 5% ionic imbalance are typically deemed acceptable, while discrepancies exceeding 10% may suggest anomalies in measurement or incomplete data. Hence, the present analysis can be used as a validation for most of the NAWA FRACHT measurements.

### Distance between measurement stations

Regarding the distance between the streamflow measurement gauges and the NAWA FRACHT (**foen_nawaf_dist**), 16% of the stations (4 out of 24) were more than 5 km away from the respective CAMELS-CH outlet. The maximum distance is 10 km for station 2068, located in the very south of Switzerland (on the Ticino River), with an overall catchment area of 1,613.3 km^2^. Furthermore, for NAWA TREND (**foen_nawat_dist**) 14% of the stations (10 out of 72) were more than 5 km away from the CAMELS-CH outlet. Only two gauges had a distance greater than 10 km between the gauge and the sampling location. The station with the maximum distance (20 km) is 2288, located at the Rhine River, and with an overall catchment area of more than 11,000 km^2^.

Although the results suggest that for most of the stations, the distance between the discharge station and the water chemistry measurement locations of either NAWA FRACHT or NAWA TREND tend to be low, users can refer to the respective variable indicating this distance when deciding whether or not to use the station in their analysis with the CAMELS-CH data. Finally, for stations where the derived catchment area is considerably different, we suggest users to use the **q_nawat_corrector** to correct the discharge data.

### Isotope measurements validation

The isotopic composition of water samples, specifically δ ^2^H and δ ^18^O, was analyzed to assess potential deviations from the Global Meteoric Water Line (GMWL). The GMWL serves as a reference for the isotopic compositions of meteoric water, following Eq. (1). This step was included in the CAMELS-CH-Chem validation phase to demonstrate the usability of the collected data for future users.

1 $$\delta {\rm{H}}2=8.0\,\delta {\rm{O}}18+10\textperthousand $$

where δ ^2^H is the deuterium fraction and δ ^18^O the Oxygen-18. Both measurements are in permille (‰) notation according to VSMOW.

**Fig. 7:**
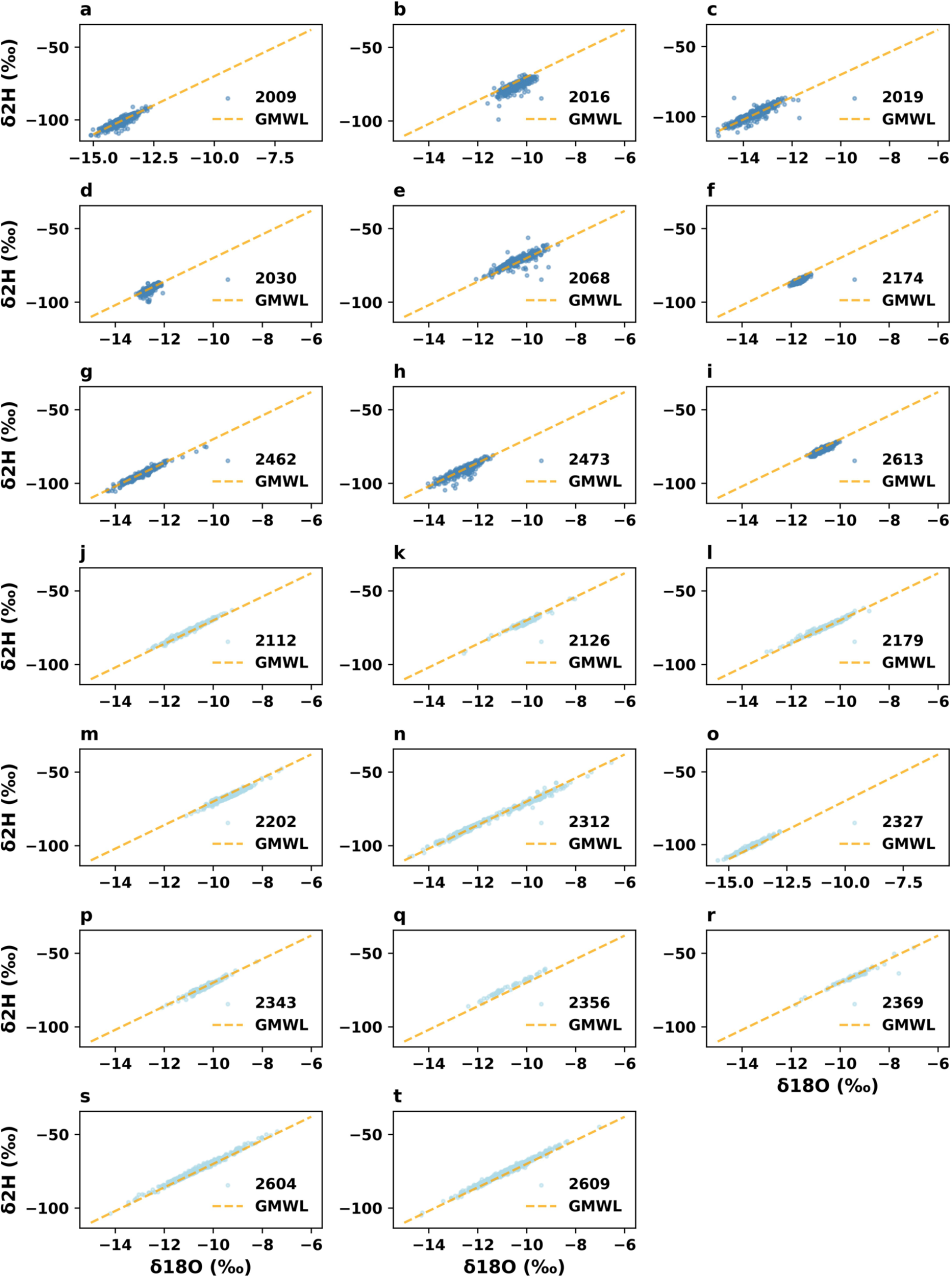
Dual isotope plots of δ²H and δ¹⁸O for the nine ISOT and 11 CH-IRP sampling locations in reference to the Global Meteoric Water Line (GMWL, dashed orange line). Blue circles (dark for ISOT and light for CH-IRP) represent individual water samples covering their respective entire timeseries.

Figure 7 shows the individual subplots **a** to **t** for the total 20 stations with isotope data (nine from ISOT and 11 from CH-IRP), with the δ²H and δ¹⁸O values plotted alongside the GMWL. The axis limits were set based on the observed range of isotope values across all stations. This comparative approach allowed for a clear identification of any deviations from the GMWL and provided insight into potential fractionation processes in the catchments.

Overall, the alignment of the isotope data with the GMWL showed low deviation, with only few samples with apparent evaporative fractionation, indicating that this dataset provides a reliable basis for future hydrological studies in the provided catchments. For CH-IRP, users can also refer to their original publication, where Staudinger *et al*.^18^ also provide lab standards and errors for their measurement stations, along with potentially problematic measurements.

## Usage Notes

Users should note that overlaps between the high-resolution and grab samples variables result from differences in temporal aggregation of the same measurement. Moreover, missing data was generally sparse and occurred sporadically, primarily due to temporary sensor or sampling interruptions, and users should acknowledge that while using the data.

Other water quality and isotope datasets for stations not covered in the current version of the CAMELS-CH-Chem dataset are available. We provide an initial, non-exhaustive list of published and unpublished datasets. This resource is intended to be expanded through ongoing contributions from the community.

## Supplementary information

Supplementary Material to: “Swiss data quality: augmenting CAMELS-CH with isotopes, water quality, agricultural and atmospheric data”

## Data Availability

The code used to produce the current dataset is available at: https://github.com/camels-ch/camels-ch-chem. The scripts are organized to enable users to follow a logical sequence during code usage. Finally, the code used to derive all figures and the technical validation is available at https://github.com/thiagovmdon/camels-ch-chem-paper.
