# Supplementary material for: Swiss data quality: augmenting CAMELS-CH with isotopes, water quality, agricultural and atmospheric data
Source: Sci Data. 2025 Jul 23;12:1283. doi: 10.1038/s41597-025-05625-1 (PMC12287458; doi:10.1038/s41597-025-05625-1)
Supplement: Supplementary file 1 — Supplementary Material to: “Swiss data quality: augmenting CAMELS-CH with isotopes, water quality, agricultural and atmospheric data” [file 41597_2025_5625_MOESM1_ESM.docx]

**Supplementary Material to: “Swiss data quality: augmenting CAMELS-CH with isotopes, water quality, agricultural and atmospheric data”**

Authors

Thiago V. M. do Nascimento^1,2^, Marvin Höge^1^, Ursula Schönenberger^1^, Sandra Pool^1^, Rosi Siber^1^, Martina Kauzlaric^3,5^, Maria Staudinger^2^, Pascal Horton^3,5^, Marius G. Floriancic^4^, Florian R. Storck^6^, Päivi Rinta^6^, Jan Seibert^2^ and Fabrizio Fenicia^1^

Affiliations

1. Eawag: Swiss Federal Institute of Aquatic Science and Technology, Dübendorf, Switzerland

2. Department of Geography, University of Zurich, Zurich, Switzerland

3. Geographisches Insitut, Universität Bern, Bern, Switzerland

4. ETH Zürich, Zurich, Switzerland

5. Oeschger Centre for Climate Change Research, University of Bern, Bern, Switzerland

6. Hydrology Division, Federal Office for the Environment, 3003 Bern, Switzerland

corresponding author: Thiago Nascimento (thiago.nascimento@eawag.ch)

**Supplementary material**

**Table of Contents**

[High-resolution measurements data 1](#_Toc201043389)

[NAWA FRACHT (previously called NADUF) 2](#_Toc201043390)

[NAWA TREND 3](#_Toc201043391)

[Ionic balance of NAWA FRACHT data 4](#_Toc201043392)

[References 5](#_Toc201043393)

## High-resolution measurements data

Table S 1. Overview of the manufacturer type, accuracy and general remarks for the intruments usef for obtainining the high-resolution measurements data. Information available at FOEN^1^ (https://www.bafu.admin.ch/dam/bafu/de/dokumente/hydrologie/fachinfo-daten/naduf_verwendetemesselektroden.pdf.download.pdf/NAWA%20FRACHT_verwendete%20Messelektroden_2025-DE.pdf).

| Name of attribute | Years | Manufacturer/Type | Accuracy according to manufacturer | Remarks |
| --- | --- | --- | --- | --- |
| temp_sensor | 1976 -1991 | Hartmannn u. Braun / CMR TEUC with PT100 (3 conductors) | 0.2°C | - |
|  | since 1991 | Camille Bauer / Eurax V601 with PT100 (3 Leiter) | 0.15 + 0.02 °C/°C | - |
|  | since 1991 | Camille Bauer / Eurax V604 mit PT100 (3 conductors) | 0.15 + 0.02 °C/°C | - |
|  | since 2002 | Rotax PT100 3-conductors resistance thermometer | 0.15 + 0.02 °C/°C | - |
|  | since 2011 | Rotax PT100 3-conductors resistance thermometer | 0.15 + 0.02 °C/°C | - |
|  | since 2018/19 | Rotax PT100 4-conductors resistance thermometer | 0.1°C +0.0017 °C/°C | - |
| ec_sensor | 1976 - 1991 | Wösthoff / EMHD 1 | 5 - 10 μS/cm | - |
|  | since 1991 | Siemens / SIPAN 4EL | 4 - 7 μS/cm | - |
|  | since 2002 | Quadroline LF296 with Tetracon 700 | 0.5% + 1Digit | - |
|  | since 2018/19 | Endress+Hauser / Memosens CLS82D | < 4 % | - |
|  | since 2022 | WTW TetraCon 700 IQ | < 4 % | only  Andelfingen hydro_id_2044,  Rekingen hydro_id_2143, Weil hydro_id_2613 |
| O2C_sensor | 1976 - 1981 | WTW / OX1 39 | 0.23 mg/l | - |
|  | 1981 - 1991 | Orbisphère / Modèle 2116 | 0.2 mg/l | - |
|  | 2005 /2006 | Hach-Lange / LDO oxygen measurement | ± 0.2 mg/l | - |
|  | since 2018/19 | Endress+Hauser / Oxymax COS61D | ± 2 % | - |
| pH_sensor | 1976 - 1991 | Hartmann u. Braun / UPY3 | 0.02 | - |
|  | since 1990 | Jenco / Modell 6300N | 0.10% | - |
|  | since 1993 | Endress+Hauser / Mycom CPM 121 mit Ceratex CPS 31 | 0.03 - 0.04 | - |
|  | since 2018/19 | Endress+Hauser / Memosens CPS31D | < 0.05 | - |

## NAWA FRACHT (previously called NADUF)

Table S 2. Overview of the methods and intruments accuracy used obtaining the NAWA FRACHT data, and available at FOEN^2^ (https://www.bafu.admin.ch/dam/bafu/de/dokumente/hydrologie/fachinfo-daten/naduf-methoden-chemische-analysen-eawag-2018.pdf.download.pdf/Methoden_NAWA%20FRACHT_2025_DE.pdf).

| Name of attribute | Units | Method | min loq | max loq |
| --- | --- | --- | --- | --- |
| alk | mmol/l | acidimetric titration (automated) | 0.02 | 0.2 |
| As | µg/l | ICP-MS | 0.5 | 0.5 |
| Ba | µg/l | ICP-MS | 0.5 | 0.5 |
| Br | mg/l | IC | 0.01 | 0.05 |
| Cd | µg/l | ET AAS / ICP-MS | 0.01 | 0.02 |
| Ca | mg/l | calculated from hardness minus magnesium |  |  |
| Cl | mg/l | CFA (photometric) / IC | 0.5 | 1.5 |
| Cr | µg/l | ET AAS / ICP-MS | 0.1 | 0.5 |
| Cu | µg/l | ET AAS / ICP-MS | 0.05 | 0.5 |
| doc | mg/l | combustion-infrared / wet-oxidation-infrared | 0.1 | 0.5 |
| drp | mg/l | CFA (photometric) / photometric | 0.001 | 0.005 |
| ec25_online | µS/cm |  |  |  |
| ec20_lab | µS/cm |  |  |  |
| F | mg/l | IC | 0.01 | 0.05 |
| Fe | mg/l | ET AAS / ICP-MS | 0.1 | 0.5 |
| Pb | µg/l | ET AAS / ICP-MS | 0.1 | 0.2 |
| Mg | mg/l | AAS / ICP-AES / IC | 0.05 | 1 |
| q_online | m^3^/s |  |  |  |
| Hg | µg/l | Cold-vapour-AAS | 0.002 | 0.05 |
| Ni | µg/l | ET AAS / ICP-MS | 0.1 | 0.5 |
| NO3___N | mg/l | CFA (photometric) / IC / photometric | 0.005 | 0.2 |
| O2C_online | mg/l |  |  |  |
| O2S_online | % | calculated from oxygen concentration, water temperature and elevation |  |  |
| pH_lab | - |  |  |  |
| pH_online | - |  |  |  |
| K | mg/l | AAS / ICP-AES / IC / IC | 0.05 | 0.5 |
| H4SiO4 | mg/l | CFA (photometric) | 0.35 | 1 |
| Na | mg/l | AAS / ICP-AES / IC / IC | 0.05 | 2 |
| Sr | µg/l | ICP-MS | 5 | 5 |
| SO4 | mg/l | titration / FIA (photometric) / IC | 1 | 5 |
| tfp | mg/l | persulfate digestion | 0.003 | 0.003 |
| th | mmol/ | EDTA titration (automated) | 0.09 | 0.2 |
| tn | mg/l | persulfate digestion / combustion-chemiluminescence | 0.1 | 0.5 |
| toc | mg/l | combustion-infrared | 0.1 | 0.5 |
| tp | mg/l | hydrogen peroxide digestion / Persulfate digestion | 0.003 | 0.02 |
| tss | mg/l | filtration and weighing | 1 | 1 |
| temp_online | °C |  |  |  |
| Zn | µg/l | AAS / ICP-MS | 0.1 | 1 |

## NAWA TREND

These data are measured by cantonal authorities according to the Swiss Modular Stepwise Procedure (https://modul-stufen-konzept.ch/en/nutrients/). No detailed measurement methods are recorded, only the range of the limits of quantifications.

Table S 3. Overview of the intruments accuracy used obtaining the NAWA TREND data.

| Name of attribute | Units | min loq | max loq |
| --- | --- | --- | --- |
| NH4_N | mg/l | 0.002 | 0.096 |
| Cl | mg/l | 0.005 | 5.6 |
| q_canton_max | m^3^/s |  |  |
| q_canton_mean | m^3^/s |  |  |
| q_canton_min | m^3^/s |  |  |
| doc | mg/l | 0.1 | 1 |
| drp | mg/l | 0.001 | 0.05 |
| ec25_lab | µS/cm | 0.3 | 10 |
| ec25_online | µS/cm |  |  |
| q_online | m^3^/s |  |  |
| NO3_N | mg/l | 0.002 | 1 |
| NO2_N | mg/l | 0.0006 | 1 |
| O2_lab | mg/l | 0.1 | 0.5 |
| O2_online | mg/l |  |  |
| O2S | % |  |  |
| pH_lab | - | 1 | 1 |
| pH___online | - |  |  |
| temp_lab | °C |  |  |
| temp_online | °C |  |  |
| turbidity_online | NTU |  |  |
| tn | mg/l | 0.041 | 1 |
| tp | mg/l | 0.0008 | 1.7 |

## Ionic balance of NAWA FRACHT data

Table S 4. Ionic mass balance statistics for each of the stations with NAWA FRACHT measurements.

| **Gauge id** | **Ionic mass balance (%)** | | | | |
| --- | --- | --- | --- | --- | --- |
|  | **Mean** | **P25** | **Median** | **P75** | **Maximum** |
| 2009 | 0.435985 | -0.545902 | 0.359950 | 1.299944 | 9.570224 |
| 2016 | 1.639680 | 0.733901 | 1.594390 | 2.505173 | 9.962365 |
| 2018 | 1.205843 | 0.271829 | 1.278908 | 2.133413 | 9.509595 |
| 2044 | 2.099322 | 1.391718 | 2.050711 | 2.778935 | 7.854155 |
| 2067 | 0.398352 | -1.059095 | 0.216745 | 1.551320 | 9.531689 |
| 2068 | 1.320987 | 0.111855 | 1.115087 | 2.224546 | 7.253196 |
| 2085 | 1.191098 | 0.441571 | 1.173093 | 1.783882 | 5.560173 |
| 2106 | 2.179472 | 1.479517 | 2.168280 | 2.797340 | 9.767008 |
| 2112 | 0.522289 | -0.073699 | 0.377360 | 1.071219 | 2.554439 |
| 2130 | 1.289813 | 0.740609 | 1.562212 | 2.164777 | 7.019292 |
| 2135 | 1.844861 | 1.389279 | 1.911113 | 2.438441 | 3.879754 |
| 2143 | 1.321940 | 0.553701 | 1.265323 | 2.021457 | 14.776865 |
| 2174 | 0.628014 | -0.233800 | 0.510192 | 1.335572 | 6.819916 |
| 2179 | 1.292728 | 0.817671 | 1.239803 | 1.714320 | 2.932693 |
| 2243 | 1.544782 | 0.565584 | 1.515227 | 2.398968 | 10.058679 |
| 2386 | 2.531875 | 2.043512 | 2.423313 | 3.009654 | 4.126190 |
| 2415 | 3.148087 | 2.032618 | 2.854008 | 4.268473 | 11.073515 |
| 2462 | -0.064403 | -0.901463 | -0.148776 | 0.720982 | 2.899675 |
| 2467 | 1.449764 | 0.803557 | 1.405023 | 2.055323 | 5.985865 |
| 2473 | 0.587801 | -0.299268 | 0.457965 | 1.363267 | 8.609398 |
| 2608 | 1.311412 | 0.905194 | 1.240102 | 1.643632 | 2.809975 |
| 2613 | 1.329235 | 0.510777 | 1.241231 | 2.178189 | 5.253940 |

## References

1. UFAM, B. für U. B. | O. fédéral de l’environnement O. | U. federale dell’ambiente. Im Rahmen des NAWA-FRACHT-Programms verwendete Sonden. (2025).

2. UFAM, B. für U. B. | O. fédéral de l’environnement O. | U. federale dell’ambiente. NAWA FRACHT: Methoden der chemischen Analysen. (2025).
